# Caloric restriction preserves memory and reduces anxiety of aging mice with early enhancement of neurovascular functions

**DOI:** 10.18632/aging.101094

**Published:** 2016-11-08

**Authors:** Ishita Parikh, Janet Guo, Kai-Hsiang Chuang, Yu Zhong, Ralf G. Rempe, Jared D. Hoffman, Rachel Armstrong, Björn Bauer, Anika M.S. Hartz, Ai-Ling Lin

**Affiliations:** ^1^ Sanders-Brown Center on Aging, University of Kentucky, Lexington, KY 40536, USA; ^2^ Department of Pharmacology and Nutritional Sciences, University of Kentucky, Lexington, KY 40536, USA; ^3^ Queensland Brain Institute and Centre for Advanced Imaging, University of Queensland, Brisbane, QLD 4072, Australia; ^4^ Department of Pharmaceutical Sciences, University of Kentucky, Lexington, KY 40536, USA; ^5^ Department of Biomedical Engineering, University of Kentucky, Lexington, KY 40506, USA

**Keywords:** caloric restriction, magnetic resonance imaging (MRI), cerebral blood flow, blood-brain barrier, mammalian target of rapamcyin (mTOR), cognition, anxiety

## Abstract

Neurovascular integrity plays an important role in protecting cognitive and mental health in aging. Lifestyle interventions that sustain neurovascular integrity may thus be critical on preserving brain functions in aging and reducing the risk for age-related neurodegenerative disorders. Here we show that caloric restriction (CR) had an early effect on neurovascular enhancements, and played a critical role in preserving vascular, cognitive and mental health in aging. In particular, we found that CR significantly enhanced cerebral blood flow (CBF) and blood-brain barrier function in young mice at 5-6 months of age. The neurovascular enhancements were associated with reduced mammalian target of rapamycin expression, elevated endothelial nitric oxide synthase signaling, and increased ketone bodies utilization. With age, CR decelerated the rate of decline in CBF. The preserved CBF in hippocampus and frontal cortex were highly correlated with preserved memory and learning, and reduced anxiety, of the aging mice treated with CR (18-20 months of age). Our results suggest that dietary intervention started in the early stage (e.g., young adults) may benefit cognitive and mental reserve in aging. Understanding nutritional effects on neurovascular functions may have profound implications in human brain aging and age-related neurodegenerative disorders.

## INTRODUCTION

Neurovascular functions, including cerebral blood flow (CBF) and blood-brain-barrier (BBB) function, play an important role on determining cognitive capability and mental health [1]. Studies have shown that neurovascular risk is highly associated with accelerated decline in language ability, verbal memory, attention and visuospatial abilities [2, 3]. Reduced CBF is linked to anxiety and depression [4–6], and impaired BBB is associated with neuroinflammation and synaptic dysfunction [7]. These neurovascular deficits are exacerbated with age [8] and in a more rapid and profound fashion in neurodegenerative disorders, including Alzheimer's disease (AD) [9–12]. Interventions that are able to maintain neurovascular integrity are thus considered crucial for impeding age-related neurological disorders.

Caloric restriction (CR), without malnutrition, is the most studied intervention that has been shown to extend the longevity of a broad range of species [13–15]. In the central nervous system, CR has also been shown to induce anti-inflammatory mechanism, reduce oxidative stress and promote synaptic plasticity [16]. In aging, CR protects mitochondrial function, neuronal activity, brain volume size and white matter integrity [14, 17, 18]. Enhanced memory in elderly humans and aging animals has also been reported with CR [19–22]. In animal models of AD, CR reduces amyloid beta (Aβ) deposition and preserves memory [23, 24]. However, the impact of CR on CBF and BBB, and the interplay between *in vivo* neurovascular functions, cognitive aging, and mental health, remain unknown.

In this study, our primary goal was to identify age-related changes of neurovascular integrity in response to CR. We previously showed that CR is protective for CBF in old adult rodents [25]. Here we further determined whether CR shows early effects on neurovascular functions, and the potential changes in vascular signaling markers thereof, in young adult animals. Our secondary goal was to determine the correlation between neurovascular function, cognitive integrity, and mental health across the young and old mice. We hypothesized that CR has significantly protective effects on CBF and BBB, which may contribute to preserved neurovascular integrity, learning and memory, and reduced anxiety in aging. We used magnetic resonance imaging (MRI) to quantify *in vivo* CBF and confocal imaging to measure BBB function, and biochemical assays to determine neurovascular signaling markers. Behavioral tests were used to assess cognition, anxiety of the mice, and the correlation between behavioral and neurovascular outcomes. The findings from this study will enhance our understanding regarding the effectiveness of nutritional intervention on brain functions in aging.

## RESULTS

### Caloric restriction enhances neurovascular functions in young mice

**Figure 1:**
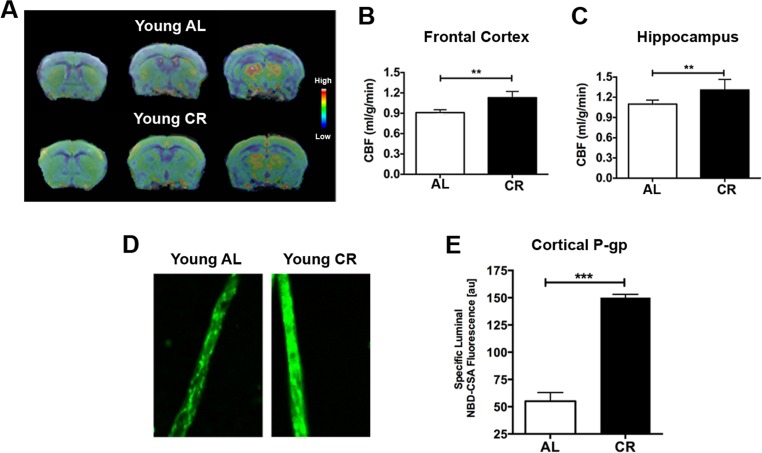
Caloric restriction enhances neurovascular functions in young mice (**A**) CBF maps superimposed on structural brain images; the color code indicates the level of CBF in a linear scale. Quantitative CBF (ml/g/min) obtained from (**B**) Frontal Cortex and (**C**) Hippocampus. (**D**) Representative confocal images showing increased luminal accumulation of NBD-CSA fluorescence (green) in brain capillaries isolated from young CR mice; shown in arbitrary fluorescence units (scale 0-255). (**E**) Corresponding quantitative fluorescence data. Data are mean ± SEM. **p < 0.01; ***p < 0.001; n.s.: non-significant; AL: ad libitum; CR: caloric restriction.

We firstly determined the CBF and BBB changes in response to CR in young adult mice (5-6 months of age). We used MRI to measure CBF in mice fed with either *ad libitum* (AL) or 40% CR (N = 12 per group). Fig. 1A shows the group-averaged CBF images of AL and CR mice. The CBF level is colorized in a linear scale, indicating that CR mice have overall higher CBF compared to the AL mice. We did further CBF analyses in brain regions associated with cognitive functions (e.g., memory and learning) based on MRI structural imaging and mouse brain atlas. We found that young CR mice had significantly higher CBF in frontal cortex (*p* < 0.01; Fig. 1B) and hippocampus (*p* < 0.01, Fig. 1C), compared to young AL mice.

BBB function was determined by measuring P-glycoprotein (P-gp) transport activity from cortical capillaries. P-gp is an ATP-driven transporter highly expressed at the BBB that facilitates clearance of Aβ, a hallmark of AD. We previously established a confocal imaging-based assay to assess P-gp transport activity in freshly isolated brain capillaries from mice [26, 27]. This assay measures within capillary lumens accumulation of [N-ε(4-nitro-benzofurazan-7-yl)-D-Lys(8)]-cyclosporin A (NBD-CSA), a fluorescent P-glycoprotein substrate. Fig. 1D shows representative confocal images of capillaries incubated to steady state in medium containing 2 μM NBD-CSA; the intensity of fluorescence in the capillary lumen reflects the amount of NBD-CSA transported by P-gp. The corresponding quantitative results are shown in Fig. 1E. Young CR mice had enhanced P-gp transport activity (2.4 fold increase; *p* < 0.0001) compared to AL mice.

### Caloric restriction enhances vascular signaling markers and shifts metabolism in young mice

**Figure 2:**
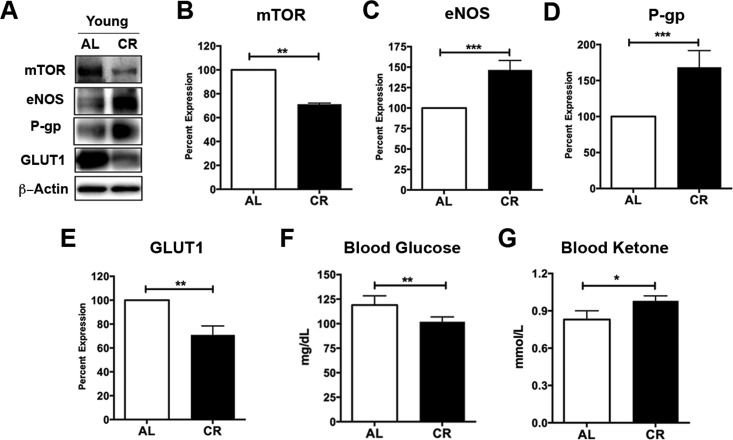
Caloric restriction enriches vascular signaling markers and shifts metabolism in young mice (**A**) Western blotting (WB) of mTOR, eNOS, P-gp and GLUT1 from the cortical vasculature, β-Actin was used as loading control; corresponding values of (**B**) mTOR, (**C**) eNOS, (**D**) P-gp, and (**E**) GLUT1 between the young AL and CR mice. All the WB data were normalized to β-Actin and compared to young AL (100%). (**F**) Blood glucose and (**G**) Blood Ketone levels of the mice. *p < 0.05; **p < 0.01; ***p < 0.001; AL: ad libitum; CR: caloric restriction.

Caloric restriction has been shown to inhibit mammalian target of rapamycin (mTOR), a nutrient sensor, in response to cellular energy status and growth factors [28, 29]. We and others have previously showed that inhibiting mTOR signaling activates endothelial nitric oxide synthase (eNOS) and releases nitric oxide, a vasodilator, which in turn causes increased CBF [30, 31]. To determine whether the enhancement of neurovascular functions in the young adult mice is also associated with mTOR signaling, we measured the protein levels of mTOR and eNOS in capillaries isolated from young CR and AL mice (Fig. 2A). We found that, compared to AL mice, CR mice had significantly lower level of mTOR (decrease to 71.1 ± 1% over 100% controls; *p* < 0.01; Fig. 2B), but higher level of eNOS (increase by 146.5 ± 11.6% over 100% controls; *p* < 0.001; Fig. 2C), consistent with our previous findings [30]. We also measured P-gp protein expression levels (Fig. 2A). Similar to the results of P-gp activity, we found that CR mice had significant enhancement in P-gp protein levels compared to the AL mice (increase by 168.4 ± 23.1% over 100% controls; *p* < 0.001; Fig. 2D).

Reduced mTOR also implies metabolic status changes, we thus measured levels of the glucose transporter 1 (GLUT1) using Western blot (Fig. 2A). We observed a significant decrease of GLUT1 in young CR mice compared to the young AL mice (decrease to 70.9 ± 7.5% over 100% controls; *p* < 0.01; Fig. 2E). This is consistent with blood glucose results, showing that CR mice had significantly reduced blood glucose levels relative to the AL mice (*p* < 0.01; Fig. 2F). In contrast, CR mice had significantly higher levels of blood ketone bodies compared to the AL mice (*p* < 0.05, Fig. 2G). This is consistent with our previous findings that caloric restriction shifts metabolism from glucose to ketone bodies utilization [17, 25].

### Caloric restriction decelerates the rate of decline of cerebral blood flow in aging mice

**Figure 3:**
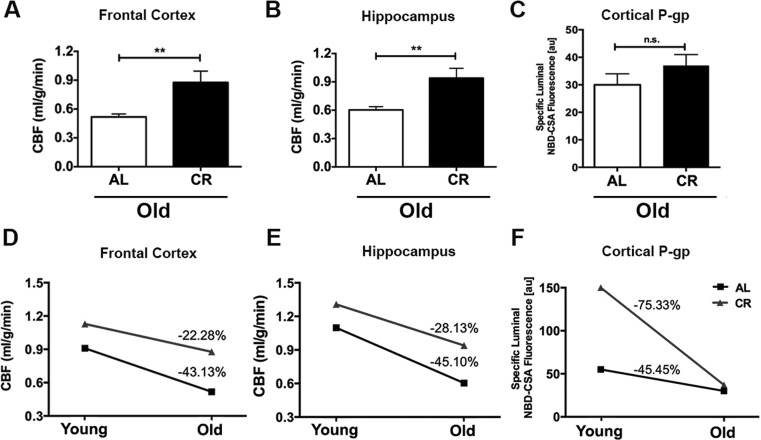
Caloric restriction decelerates the rate of decline of cerebral blood flow in aging mice Old CR mice had significantly higher CBF in (**A**) Frontal cortex and (**B**) Hippocampus; however (**C**) P-gp activity did not show significance when compared with old AL mice. The age-dependent changes between AL and CR mice in (**D**) CBF within frontal cortex, and (**E**) hippocampus, and (**F**) *Difference in* cortical P-gp activity, between AL and CR mice. Data are mean ± SEM. **p < 0.01; n.s.: non-significant; AL: ad libitum; CR: caloric restriction.

We further determined the CBF and BBB function changes in response to CR in old adult mice (18-20 months of age; 12 AL and 12 CR mice). Similar to the findings in the young mice, we found that old CR mice had significantly higher CBF compared to the old AL mice, both in the frontal cortex (*p* < 0.01; Fig. 3A) and hippocampus (*p* < 0.01, Fig. 3B). However, we did not observe a difference in P-gp activity between the two groups (*p* > 0.05, Fig. 3C).

We calculated the age-related changes in CBF and BBB function. We found that in the frontal cortex, old AL mice had 43.13% group averaged decline of CBF compared to the young AL mice (no variation was available due to cross-sectional comparison); In contrast, the old CR mice had only 22.28% averaged reduction compared to the young CR mice (Fig. 3D).

Similar pattern was found in hippocampus – old AL mice had 45.10% averaged decreases in CBF, whereas old CR mice had 28.13% averaged reduction, compared to their young littermates (Fig. 3E). On the other hand, we found that there was a higher reduction rate of P-gp activity in the CR group (−75.33%) compared with the AL group (−45.45%; Fig. 3F). Collectively, we found that caloric restriction impeded the age-dependent decline of CBF but not P-gp activity (BBB function).

### Caloric restriction preserves learning and long-term memory of aging mice

**Figure 4:**
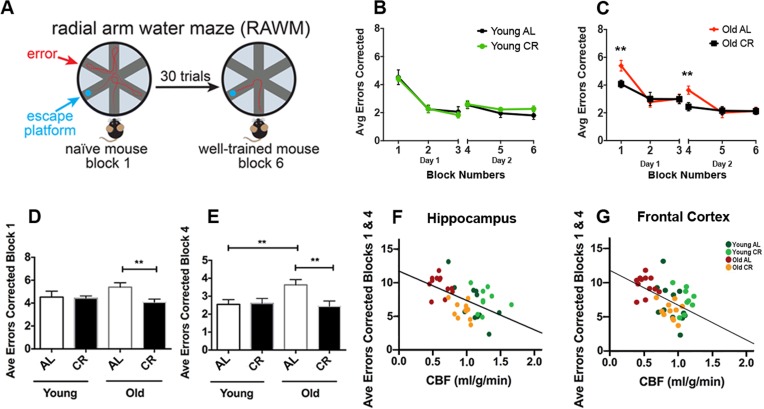
Caloric restriction preserves learning and long-term memory of aging mice (**A**) An illustration of the Radial Arm Water Maze. Mice were trained to locate the hidden platform through a 2-day, 30 trials testing (3 blocks each day, 5 trials each block). Wrong entries were recorded as errors. (**B**) Young AL and CR mice did not have significantly different performances across the 6 blocks. (**C**) Old AL mice performed worse in Block 1 (initial learning phase) and Block 4 (initial recall phase) compared to the old CR mice. The comparison among the four groups for (**D**) Block 1 and (**E**) Block 4. Significant inverse correlation between errors made in Blocks 1 and 4 and CBF in (**F**) hippocampus (r^2^ = 0.29, *p* < 0.001) and (**G**) frontal cortex (r^2^ = 0.27, *p* < 0.001). Color codes indicate the four groups of mice. Data are mean ± SEM. **p < 0.01; AL: ad libitum; CR: caloric restriction.

We used radial arm water maze (RAWM) to evaluate learning and spatial memory of the mice [32, 33]. Fig. 4A illustrates the RAWM assessment. The task for the mice was to identify the arm which contains a hidden platform. Wrong entries of the arms were recorded as errors. The protocol consisted of a two-day testing paradigm. Day 1 was the “learning” phase where mice went through three blocks (Blocks 1-3; 5 trials in each block) to test learning and short-term spatial memory. Day 2 was the “recall” phase where mice went through three additional blocks (Blocks 4-6) to test long-term memory after a 24-hour retention period to locate the platform. It is expected that after the two-day training, the mouse with intact memory could find the platform with minimal errors. In young mice, we saw no significant difference between AL and CR groups (Fig. 4B). In old mice, however, AL group made significantly more errors in the initial learning phase (Block 1; *p* < 0.01) and the initial recall phase (Block 4; *p* < 0.01), compared to the CR group (Fig. 4C). In addition, we found that old CR had similar performances in both Block 1 (Fig. 4D; *p* > 0.05) and Block 4 (Fig. 4E; *p* > 0.05) compared to the young mice (both AL and CR), suggesting that old CR mice had preserved learning and long-term memory abilities.

We used combined errors from Blocks 1 and 4 to further identify the correlation between cognitive function and CBF. We found that the errors made in RAWM had significantly inverse correlations with hippocampal CBF (r^2^ = 0.29, *p* < 0.001; Fig. 4F) and frontal CBF (r^2^ = 0.27, *p* < 0.001; Fig. 4G), indicating that level of CBF in cognition-associated brain regions is highly associated with learning and spatial memory performances.

### Caloric restriction reduces anxiety of aging mice

**Figure 5:**
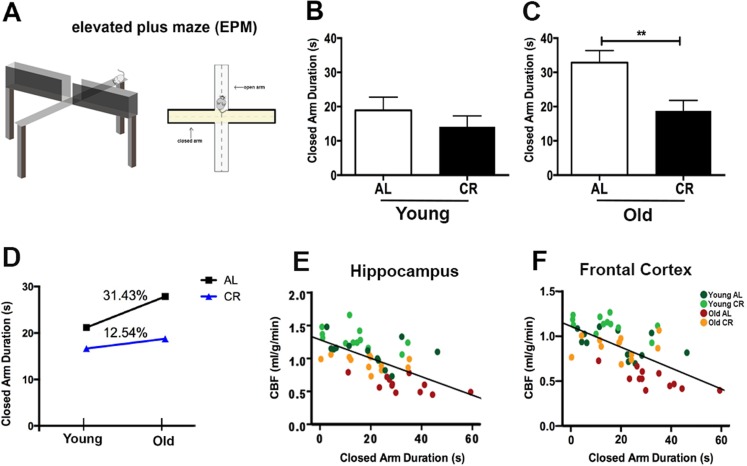
Caloric restriction reduces anxiety of aging mice (**A**) An illustration of the Elevated Plus Maze. The maze consists of four arms (two enclosed arms and two open arms) elevated 100 cm above the floor. Anxiety level was determined the time spent in the closed arms (conceived as a safe place) over a 5 minutes testing session. Closed arm duration (in seconds) of (**B**) Young AL and CR mice, and (**C**) Old AL and CR mice. (**D**) The age-dependent changes of anxiety level between AL and CR mice. Significant inverse correlation between closed arm duration and CBF in (**E**) hippocampus (r^2^ = 0.40, *p* < 0.0001) and (**F**) frontal cortex (r^2^ = 0.39, *p* < 0.0001). Color codes indicate the four groups of mice. Data are mean ± SEM. **p < 0.01; AL: ad libitum; CR: caloric restriction.

We used elevated plus maze (EPM) to evaluate anxiety of the mice (Fig. 5A) [34]. The EPM consists of two open and two closed arms. Closed arms are perceived as safe zones, and thus mice with higher anxiety had tendency to stay in the closed arms. We determined the anxiety-related behavior by measuring the time spent in the closed arms over the 5 min. test session. For the young mice, we did not find significant differences between the CR and AL groups (*p* > 0.05; Fig. 5B), though CR mice had a trend of less time in the closed arms. In contrast, old AL mice spent significantly longer time in the closed arms compared to the old CR mice (*p* < 0.01; Fig. 5C), indicating higher anxiety of the old AL mice. Consequently, when comparing the age-related performances, the AL group showed higher increases in anxiety (31.43% group averaged) compared to the CR mice (12.54% group averaged) (Fig. 5D). We also found it had significant and inverse correlations between closed arm duration and CBF in hippocampus (r^2^ = 0.40, *p* < 0.0001; Fig. 5E) and in frontal cortex (r^2^ = 0.39, *p* < 0.0001; Fig. 5F).

## DISCUSSION

To our knowledge, this is the first study to investigate the interplay between CR-induced changes of neurovascular integrity, cognitive function, and mental health in aging using neuroimaging, behavioral assessments, and biochemical assays. In this study, we demonstrate (i) in young mice, CR enhanced CBF and BBB function, (ii) inhibited mTOR, which was associated with increased eNOS, reduced glucose metabolism, and increased blood ketone level; (iii) in the aging mice, CR preserved CBF, learning, long-term memory, and reduced anxiety; (iv) hippocampal and frontal CBF exhibited high association with cognitive performance and inversely correlated with anxiety level.

Our results indicate that young CR mice had significant enhancement of CBF and BBB function, which in turn may be associated with mTOR signaling changes induced by CR. In addition to modulating eNOS, we previously demonstrated that mTOR inhibition reduces proinflammatory cytokine that breaks down BBB, and thus restores BBB integrity and CBF in mice that carries human APOE4 gene [35]. Furthermore, mTOR is able to clean up misfolded proteins (such as Aβ and cerebral amyloid angiopathy) via regulation of autophagy [36], therefore, it can further reduce athero-sclerosis and preserve neurovascular integrity, e.g., vascular density [30]. mTOR signaling also integrates the effects of brain-derived neurotrophic factor, which works with insulin/insulin-like growth factors and serotonin, to exert beneficial effects on vascular system by decreasing blood pressure, atherogenic lipids, inflammatory cytokines and oxidative stress, and increased cellular stress resistance [37, 38]. As shown in the present and previous studies, mTOR inhibition leads to metabolic shift from utilizing carbohydrate (e.g., glucose) to fatty acid (e.g., ketone bodies produced from fatty acids) [17, 25, 39, 40]. Increased levels of ketone bodies have shown to evoke CBF response, and reduce oxidative stress, neuroinflammation and Aβ [39, 41–44].

Increased CBF and ketone bodies metabolism suggest increased oxidative metabolism within the brain of young mice. Basal CBF is tightly coupled with cerebral metabolic rate of oxygen [45] and utilization of ketone bodies significantly elevate the oxygen utilization in mitochondria through beta-oxidation of fatty acid [40, 46]. This is supported by evidence from isolated mitochondria, showing CR enhances mitochondrial function and induces bioenergetic efficiency (7,19). This is also consistent with our previous imaging findings that old animals with chronic CR diet had pre-served oxidative metabolism, mitochondrial functions (TCA cycle flux and ATP production), and neuronal activity (neurotransmission rate) compared to the old AL animals [18]. The increased oxidative metabolism, particularly in brain regions associated with cognition (e.g., frontal cortex and hippocampus), may also play a crucial role for neuronal and cognitive protections. Previous studies showed that cognition-associated brain regions have non-oxidative glycolysis exceeding the required needs of oxidative phosphorylation, a phenomenon known as aerobic glycolysis (AG) [47]. Excessive AG (or the “Warburg effect”) is a key process that sustains T cell activation and differentiation and is involved in inflammatory-mediated conditions [48]. In line with this, the distribution of AG in normal young adults is spatially correlated with Aβ deposition in AD patients and cognitively normal individuals with elevated Aβ [49, 50]. Animal studies further demonstrated that Aβ plaque formation is an activity dependent process associated with AG [49, 51]. Therefore, increased oxidative metabolism in cognition-related regions may decrease AG and thus reduce the risk for AD, consistent with the findings in CR mice [23, 24].

The early-life neurovascular and neurometabolic changes may play a critical role in protecting physiological functionality and enhancing cognitive reserve with age [52]. Recent studies reported that lifestyle factors act as moderators for cognitive reserve to protect against AD in the elderly [52, 53]. The study suggested that neuroprotective mechanisms play a major role during early stages and compensatory mechanisms in later stages of the disease. In line with this, we observed that the CR mice had a slower reduction rate in CBF with age compared with the AL mice. As a result, the old CR mice had comparable levels of CBF compared to the young AL mice, similar to our previous findings in rats [25]. The preservation of hippocampal and frontal CBF were associated with improved cognition in the old CR mice. This is also consistent with our previous findings that restoration of CBF in young mice modeling human AD could potentially prevent their cognitive decline in aging [30, 35].

We further identified that CR reduced anxiety levels in aging mice. Similar findings were reported in a recent study, showing rhesus monkeys on a 30% CR diet demonstrated less anxious behavior than controls in different arousing contexts [54]. These results are consistent with other studies, indicating that mTOR inhibition is playing an important role on anxiety- and depressive-like behaviors [55]. Treating mice with rapamycin, an mTOR inhibitor, Halloran et al. observed reduced anxiety-like behavior, e.g., reduced thigmotaxis (swimming in close proximity to the pool wall), in mice with AD-like pathology. They also found a reduction in depressive-like behavior in the mice, e.g., floating during training phase of the Morris water maze and reduced time spent immobile on the tail suspension test. Similar effects have been reported for genetically modified mice with autistic-like behavior, showing that mTOR inhibition attenuated anxiety, hyperexcitability, abnormal social interaction, repetitive behavior and vocalizations of the mice [56, 57]. It has been shown that the changes in anxiety- and depressive-like behavior were correlated with increased levels of dopamine and dopamine metabolites in the midbrain [55]. Our study found that CBF was highly correlated (inversely) with anxiety level, which is consistent with previous findings [4–6].

We demonstrated that CR had beneficial effects on aging brain functions, potentially via the mTOR pathway. Genetic or pharmacological inhibition of mTOR signaling has shown to slow aging and extend lifespan in various species, and confer protection against many age-related pathologies [58]. Here we further show that activating the mTOR pathway could potentially protect brain function (both cognitive and non-cognitive types of behavior) in healthy aging by preserving neurovascular function. Specifically, mTOR inhibition was associated with increased eNOS, which may contribute to CBF enhancements in young CR mice and preservation in old CR mice. In AL mice (control), we found decreased P-gp protein expression and activity with age, which is consistent with previous literature findings in different mouse models [59–61]. In CR mice, however, calorie restriction did not preserve P-gp expression and activity with age. To this date it is unknown how blood-brain barrier P-gp activity is regulated in aging and how it can be preserved. Thus, more studies are needed to investigate potential signaling steps that lead to age-mediated reduction of P-gp at the BBB. Future studies will also need to further investigate the potential mechanism of CR effects on BBB function with age.

It has to be pointed out that we used a long-lived animal model (C57BL/6N) in the study. We recognize, however, that the CR effects may be strain- and genetics- dependent. Recent studies showed that CR had adversely impacted several inbred mice, including shortening lifespan and impairing fat storage with age [62, 63]. Therefore, it would be important for future studies to take into account genetic background to identify the effects of CR on brain aging. This is also applicable for humans, where each individual would have different responses to identical foods due to their genotype. Recent studies have shown that personalized diet could be prescribed based on the individual's genetic response to post-meal blood sugar changes [64]. Precision nutrition would be very useful in the future to slow down brain aging and to prevent AD, and the progress can be monitored by in vivo neuroimaging and cognitive testing.

In conclusion, we used neuroimaging, behavioral assessments and biochemical assays to identify correlations between vascular integrity, cognitive functions, and mental health induced by CR in aging mice. We showed that neurovascular functions were enhanced in young CR mice, as well as preservation of CBF, cognition, and anxiety level in aging CR mice. Our study suggests that mTOR pathway may be critically involved in the process. These findings imply that dietary intervention started in the early stage (e.g., young adults) may benefit cognitive reserve in aging. Understanding nutritional effects on brain vascular, cognitive, and mental functions may have profound implications in human aging and other age-related neurodegenerative disorders. In the future, using in vivo MRI and cognitive assessments, we could be in a position to identify effective personalized nutritional interventions and treatment efficacy there-of to slow down brain aging and/or prevent dementia in humans.

## METHODS

### Animals

We used male C57BL/6N mice in the study as they demonstrated extended longevity with CR [65, 66]. Young (5–6 months) and old (18-20 months) adult mice were obtained from the National Institute on Aging Caloric Restriction Colony. At the National Institute on Aging, all mice were fed *ad libitum* [National Institutes of Health (NIH)-31 diet] until 14 weeks of age. The CR regimen was initiated by incremental caloric reduction of 10% at week 14, 25% at week 15, and reaching full 40% CR by week 16 with continuation of the diet over the lifetime. The vitamin-fortified NIH-31 (NIH-31 fortified) diet fed to CR mice provided 60% of the calories and additional vitamins supplement consumed by *ad libitum* mice. After arriving at our facilities, mice were housed individually (1 mouse per cage) in a specific pathogen-free facility. We determined the sample size with power analysis in order to perform the comparison at a 0.05 level of significance, with a 90% chance of detecting a true difference of all the measurements between the four groups. Twelve mice per group were used in the study. All experimental procedures were approved by the Institutional Animal Care and Use Committee (IACUC) at the University of Kentucky (UK) and in compliance with the ARRIVE guidelines [67].

### Cerebral blood flow measurement

MRI experiments were performed on a 7T MR scanner (Clinscan, Bruker BioSpin, Germany) at the Magnetic Resonance Imaging & Spectroscopy Center of the University of Kentucky. Mice were anesthetized with 4.0% isoflurane for induction and then maintained in a 1.2% isoflurane and air mixture using a nose cone. Heart rate (90–110 bpm), respiration rate (50-80 breaths/min), and rectal temperature (37 ± 1°C) was continuously monitored and maintained. T2-weighted structural images were acquired with field of view (FOV) =18 x18 mm^2^, matrix = 256 × 256; slice thickness = 1 mm, 10 slices, repetition time (TR) = 1500 ms, and echo time (TE) = 35 ms. Quantitative CBF (with units of mL/g per minute) was measured using MRI-based pseudo-continuous arterial spin labeling (pCASL) techniques. A whole body volume coil was used for transmission and a mouse brain surface coil was placed on top of the head for receiving. Paired control and label images were acquired in an interleaved fashion with a train of Hanning window-shaped radiofrequency pulses of duration/spacing = 200/200 μs, flip angle = 25° and slice-selective gradient = 9 mT/m, and a labeling duration = 2100 ms [68]. The images were acquired by 2D multi-slice spin-echo echo planner imaging with FOV =18 x18 mm^2^, matrix =128 × 128, slice thickness = 1 mm, 10 slices, TR = 4,000 ms, TE = 35 ms, and 120 repetitions. pCASL image analysis was employed with in-house written codes in MATLAB (MathWorks, Natick, MA) to obtain quantitative CBF [69].

### Radial arm water maze

As shown in Fig. 3A, the RAWM task can be used to measure both spatial working memory and spatial reference memory [32, 33]. The RAWM task was conducted in the Rodent Behavioral Core (RBC) of UK as described previously [17], following a 2-day testing paradigm. A staggered training schedule was used, running the mice in cohorts of ten mice, while alternating the different cohorts through the trials over day 1 and day 2 of the test. This alternating protocol was used to avoid the learning limitations imposed by massed sequential trials and to avoid fatigue that may result from consecutive trials. Geometric extra-maze visual cues were fixed throughout the study on three sides of the curtains. Visual platform trials were included in the training, and were used to determine if visual impairment could be a cofounding variable. The mouse performance was recorded by EthoVision XT 8.0 video tracking software (Noldus Information Technology). Data were analyzed by the EthoVision software for the number of incorrect arm entries, which are defined as errors. The video was reviewed for each mouse to ensure that the mice did not employ nonspatial strategies, such as chaining, to solve the task.

### Elevated plus maze

The EPM was also performed at RBC of UK. The maze consists of four arms (two enclosed arms and two open arms) elevated 100 cm above the floor (Fig. 5A). The time that mouse spent in the closed arms and open arms of the maze were recorded automatically over the 5 min test session by EthoVision XT 8.0 video tracking software (Noldus Information Technology).

### Blood-brain barrier function determination and Western blotting

#### Capillary isolation

Brain capillaries were isolated from mice according to a previously described protocol [26, 70]. Briefly, mice were euthanized by CO_2_ inhalation and decapitated; brains were immediately harvested and collected in ice-cold DPBS buffer supplemented with 5 mM D-glucose and 1 mM Na-pyruvate, pH 7.4. Brains were dissected by removing meninges, choroid plexus and white matter, and homogenized in DPBS. The brain homogenate was mixed with Ficoll^®^ and centrifuged at 5,800g for 20 min at 4°C. The capillary pellet was resuspended in 1% BSA buffer and first passed through a 300 μm nylon mesh and then through a 27 μm nylon mesh. Capillaries retained by the 27 μm nylon mesh were collected and washed with DPBS buffer, and used for experiments.

#### P-glycoprotein transport activity

Isolated capillaries were incubated for 1 h at room temperature with 2 μM NBD-CSA (custom-synthesized by R. Wenger (Basel, Switzerland)) in DPBS buffer. Ten capillary images were acquired by confocal microscopy (Leica TSP SP5 Confocal Microscope with Environmental Chamber, 63 × D-Water UV objective, numerical aperture 1.2, 488-nm line of an argon laser, Leica Microsystems). Confocal images were analyzed by quantitating luminal NBD-CSA fluorescence with Image J software (v.1.45s; Wayne Rasband, NIH). Specific, luminal NBD-CSA fluorescence was taken as the difference between total luminal fluorescence and fluorescence in the presence of the P-glycoprotein-specific inhibitor PSC833 (5 μM, Novartis, Basel, Switzerland) [71].

#### Western blotting and quantification

To determine protein expression, isolated brain capillaries were homogenized in tissue lysis buffer containing protease inhibitor cocktail. Homogenized brain capillary samples were centrifuged at 10,000 g for 15 min at 4°C, followed by a centrifugation of the denucleated supernatants at 100,000 g for 90 min at 4°C. Pellets (crude brain capillary plasma membranes) were resuspended and protein concentrations were determined using the Bradford protein assay. Western blots were performed using the NuPage™ electro-phoresis and blotting system from Invitrogen. Blotting membranes were incubated overnight with antibody to P-gp (C219; MA1-26528, ThermoFisher, 1μg/ml), mTOR (ab134903, Abcam, 1μg/ml), GLUT1 (ab652, Abcam, 1μg/ml), and β-actin (ab8226 from Abcam, 1:1000, 1 μg/ml). Proteins were detected using SuperSignal^®^ West Pico Chemoluminescent substrate (Pierce, Rockford, IL, USA) and protein bands were visualized with a BioRad Gel Doc™ XRS imaging system.

### Blood glucose and ketone bodies measurements

When the mice were sacrificed, blood samples were collected in 500 μl lithium heparin 12.5 IU Terumo Capiject Capillary blood collection tubes (Vacutainer K2 EDTA) to avoid blood coagulation. A total of 1–2 μl of blood sample were used to measure blood glucose level using a blood glucose meter and a test strip (Clarity Plus, Boca Raton, FL, USA). Another 10 μl of blood sample was used for ketone bodies level measurement using a STAT-Site M (β-Hydro-xybutyrate) meter and a test strip (Standbio Ketosite STAT-Site M-β HB, Boerne, TX, USA).

### Statistics

Statistical analyses were performed using GraphPad Prism 6 (GraphPad, San Diego, CA, USA). All data are expressed as mean ± SEM. Results were assessed using two-way analysis of variance (ANOVA). Tukey's test was further used as a post hoc test to detect between-group differences. Values of *p* < 0.05 were considered statistically significant.

## References

[1] Zlokovic BV (2011). Neurovascular pathways to neurodegeneration in Alzheimer's disease and other disorders. Nat Rev Neurosci.

[2] Iadecola C (2004). Neurovascular regulation in the normal brain and in Alzheimer's disease. Nat Rev Neurosci.

[3] Bangen KJ, Beiser A, Delano-Wood L, Nation DA, Lamar M, Libon DJ, Bondi MW, Seshadri S, Wolf PA, Au R (2013). APOE genotype modifies the relationship between midlife vascular risk factors and later cognitive decline. J Stroke Cerebrovasc Dis.

[4] Ebmeier KP, Cavanagh JT, Moffoot AP, Glabus MF, O'Carroll RE, Goodwin GM (1997). Cerebral perfusion correlates of depressed mood. Br J Psychiatry.

[5] Gur RC, Gur RE, Resnick SM, Skolnick BE, Alavi A, Reivich M (1987). The effect of anxiety on cortical cerebral blood flow and metabolism. J Cereb Blood Flow Metab.

[6] Park J, Moghaddam B (2016). Impact of anxiety on prefrontal cortex encoding of cognitive flexibility. Neuroscience.

[7] Bell RD, Winkler EA, Singh I, Sagare AP, Deane R, Wu Z, Holtzman DM, Betsholtz C, Armulik A, Sallstrom J, Berk BC, Zlokovic BV (2012). Apolipoprotein E controls cerebrovascular integrity via cyclophilin A. Nature.

[8] Martin AJ, Friston KJ, Colebatch JG, Frackowiak RS (1991). Decreases in regional cerebral blood flow with normal aging. J Cereb Blood Flow Metab.

[9] Thambisetty M, Beason-Held L, An Y, Kraut MA, Resnick SM (2010). APOE epsilon4 genotype and longitudinal changes in cerebral blood flow in normal aging. Arch Neurol.

[10] Nagata K, Buchan RJ, Yokoyama E, Kondoh Y, Sato M, Terashi H, Satoh Y, Watahiki Y, Senova M, Hirata Y, Hatazawa J (1997). Misery perfusion with preserved vascular reactivity in Alzheimer's disease. Ann N Y Acad Sci.

[11] Bentourkia M, Bol A, Ivanoiu A, Labar D, Sibomana M, Coppens A, Michel C, Cosnard G, De Volder AG (2000). Comparison of regional cerebral blood flow and glucose metabolism in the normal brain: effect of aging. J Neurol Sci.

[12] Montagne A, Nation DA, Pa J, Sweeney MD, Toga AW, Zlokovic BV (2016). Brain imaging of neurovascular dysfunction in Alzheimer's disease. Acta Neuropathol.

[13] Choi JS, Choi KM, Lee CK (2011). Caloric restriction improves efficiency and capacity of the mitochondrial electron transport chain in Saccharomyces cerevisiae. Biochem Biophys Res Commun.

[14] Colman RJ, Anderson RM, Johnson SC, Kastman EK, Kosmatka KJ, Beasley TM, Allison DB, Cruzen C, Simmons HA, Kemnitz JW, Weindruch R (2009). Caloric restriction delays disease onset and mortality in rhesus monkeys. Science.

[15] Rahat O, Maoz N, Cohen HY (2011). Multiple pathways regulating the calorie restriction response in yeast. J Gerontol A Biol Sci Med Sci.

[16] Gillette-Guyonnet S, Vellas B (2008). Caloric restriction and brain function. Curr Opin Clin Nutr Metab Care.

[17] Guo J, Bakshi V, Lin AL (2015). Early Shifts of Brain Metabolism by Caloric Restriction Preserve White Matter Integrity and Long-Term Memory in Aging Mice. Front Aging Neurosci.

[18] Lin AL, Coman D, Jiang L, Rothman DL, Hyder F (2014). Caloric restriction impedes age-related decline of mitochondrial function and neuronal activity. J Cereb Blood Flow Metab.

[19] Fontán-Lozano A, López-Lluch G, Delgado-García JM, Navas P, Carrión AM (2008). Molecular bases of caloric restriction regulation of neuronal synaptic plasticity. Mol Neurobiol.

[20] Mattson MP (2010). The impact of dietary energy intake on cognitive aging. Front Aging Neurosci.

[21] Valdez G, Tapia JC, Kang H, Clemenson GD, Gage FH, Lichtman JW, Sanes JR (2010). Attenuation of age-related changes in mouse neuromuscular synapses by caloric restriction and exercise. Proc Natl Acad Sci USA.

[22] Witte AV, Fobker M, Gellner R, Knecht S, Flöel A (2009). Caloric restriction improves memory in elderly humans. Proc Natl Acad Sci USA.

[23] Mouton PR, Chachich ME, Quigley C, Spangler E, Ingram DK (2009). Caloric restriction attenuates amyloid deposition in middle-aged dtg APP/PS1 mice. Neurosci Lett.

[24] Schafer MJ, Alldred MJ, Lee SH, Calhoun ME, Petkova E, Mathews PM, Ginsberg SD (2015). Reduction of β-amyloid and γ-secretase by calorie restriction in female Tg2576 mice. Neurobiol Aging.

[25] Lin AL, Zhang W, Gao X, Watts L (2015). Caloric restriction increases ketone bodies metabolism and preserves blood flow in aging brain. Neurobiol Aging.

[26] Hartz AM, Miller DS, Bauer B (2010). Restoring blood-brain barrier P-glycoprotein reduces brain amyloid-beta in a mouse model of Alzheimer's disease. Mol Pharmacol.

[27] Hartz AM, Zhong Y, Wolf A, LeVine H, Miller DS, Bauer B (2016). Aβ40 Reduces P-Glycoprotein at the Blood-Brain Barrier through the Ubiquitin-Proteasome Pathway. J Neurosci.

[28] Blagosklonny MV (2010). Calorie restriction: decelerating mTOR-driven aging from cells to organisms (including humans). Cell Cycle.

[29] Heitman J, Movva NR, Hall MN (1991). Targets for cell cycle arrest by the immunosuppressant rapamycin in yeast. Science.

[30] Lin AL, Zheng W, Halloran JJ, Burbank RR, Hussong SA, Hart MJ, Javors M, Shih YY, Muir E, Solano Fonseca R, Strong R, Richardson AG, Lechleiter JD (2013). Chronic rapamycin restores brain vascular integrity and function through NO synthase activation and improves memory in symptomatic mice modeling Alzheimer's disease. J Cereb Blood Flow Metab.

[31] Cheng C, Tempel D, Oostlander A, Helderman F, Gijsen F, Wentzel J, van Haperen R, Haitsma DB, Serruys PW, van der Steen AF, de Crom R, Krams R (2008). Rapamycin modulates the eNOS vs. shear stress relationship. Cardiovasc Res.

[32] Arendash GW, King DL, Gordon MN, Morgan D, Hatcher JM, Hope CE, Diamond DM (2001). Progressive, age-related behavioral impairments in transgenic mice carrying both mutant amyloid precursor protein and presenilin-1 transgenes. Brain Res.

[33] Sood A, Warren Beach J, Webster SJ, Terry AV, Buccafusco JJ (2007). The effects of JWB1-84-1 on memory-related task performance by amyloid Abeta transgenic mice and by young and aged monkeys. Neuropharmacology.

[34] Bachstetter AD, Webster SJ, Tu T, Goulding DS, Haiech J, Watterson DM, Van Eldik LJ (2014). Generation and behavior characterization of CaMKIIβ knockout mice. PLoS One.

[35] Lin AL, Jahrling JB, Zhang W, DeRosa N, Bakshi V, Romero P, Galvan V, Richardson A (2015). Rapamycin rescues vascular, metabolic and learning deficits in apolipoprotein E4 transgenic mice with pre-symptomatic Alzheimer's disease. J Cereb Blood Flow Metab.

[36] Son S, Stevens MM, Chao HX, Thoreen C, Hosios AM, Schweitzer LD, Weng Y, Wood K, Sabatini D, Vander Heiden MG, Manalis S (2015). Cooperative nutrient accumulation sustains growth of mammalian cells. Sci Rep.

[37] Mattson MP, Wan R (2005). Beneficial effects of intermittent fasting and caloric restriction on the cardiovascular and cerebrovascular systems. J Nutr Biochem.

[38] Gómez-Pinilla F (2008). Brain foods: the effects of nutrients on brain function. Nat Rev Neurosci.

[39] Shimazu T, Hirschey MD, Newman J, He W, Shirakawa K, Le Moan N, Grueter CA, Lim H, Saunders LR, Stevens RD, Newgard CB, Farese RV, de Cabo R (2013). Suppression of oxidative stress by β-hydroxybuty-rate, an endogenous histone deacetylase inhibitor. Science.

[40] Katewa SD, Demontis F, Kolipinski M, Hubbard A, Gill MS, Perrimon N, Melov S, Kapahi P (2012). Intramyocellular fatty-acid metabolism plays a critical role in mediating responses to dietary restriction in Drosophila melanogaster. Cell Metab.

[41] Sullivan PG, Rippy NA, Dorenbos K, Concepcion RC, Agarwal AK, Rho JM (2004). The ketogenic diet increases mitochondrial uncoupling protein levels and activity. Ann Neurol.

[42] Hasselbalch SG, Madsen PL, Hageman LP, Olsen KS, Justesen N, Holm S, Paulson OB (1996). Changes in cerebral blood flow and carbohydrate metabolism during acute hyperketonemia. Am J Physiol.

[43] Linde R, Hasselbalch SG, Topp S, Paulson OB, Madsen PL (2006). Global cerebral blood flow and metabolism during acute hyperketonemia in the awake and anesthetized rat. J Cereb Blood Flow Metab.

[44] Veech RL (2004). The therapeutic implications of ketone bodies: the effects of ketone bodies in pathological conditions: ketosis, ketogenic diet, redox states, insulin resistance, and mitochondrial metabolism. Prostaglandins Leukot Essent Fatty Acids.

[45] Fox PT, Raichle ME, Mintun MA, Dence C (1988). Nonoxidative glucose consumption during focal physiologic neural activity. Science.

[46] Chowdhury GM, Jiang L, Rothman DL, Behar KL (2014). The contribution of ketone bodies to basal and activity-dependent neuronal oxidation in vivo. J Cereb Blood Flow Metab.

[47] Vaishnavi SN, Vlassenko AG, Rundle MM, Snyder AZ, Mintun MA, Raichle ME (2010). Regional aerobic glycolysis in the human brain. Proc Natl Acad Sci USA.

[48] Palmer CS, Ostrowski M, Balderson B, Christian N, Crowe SM (2015). Glucose metabolism regulates T cell activation, differentiation, and functions. Front Immunol.

[49] Vlassenko AG, Raichle ME (2015). Brain aerobic glycolysis functions and Alzheimer's disease. Clin Transl Imaging.

[50] Vlassenko AG, Vaishnavi SN, Couture L, Sacco D, Shannon BJ, Mach RH, Morris JC, Raichle ME, Mintun MA (2010). Spatial correlation between brain aerobic glycolysis and amyloid-β (Aβ) deposition. Proc Natl Acad Sci USA.

[51] Cirrito JR, Yamada KA, Finn MB, Sloviter RS, Bales KR, May PC, Schoepp DD, Paul SM, Mennerick S, Holtzman DM (2005). Synaptic activity regulates interstitial fluid amyloid-beta levels in vivo. Neuron.

[52] Whalley LJ, Staff RT, Fox HC, Murray AD (2016). Cerebral correlates of cognitive reserve. Psychiatry Res.

[53] Arenaza-Urquijo EM, Wirth M, Chételat G (2015). Cognitive reserve and lifestyle: moving towards preclinical Alzheimer's disease. Front Aging Neurosci.

[54] Willette AA, Coe CL, Colman RJ, Bendlin BB, Kastman EK, Field AS, Alexander AL, Allison DB, Weindruch RH, Johnson SC (2012). Calorie restriction reduces psychological stress reactivity and its association with brain volume and microstructure in aged rhesus monkeys. Psychoneuroendocrinology.

[55] Halloran J, Hussong SA, Burbank R, Podlutskaya N, Fischer KE, Sloane LB, Austad SN, Strong R, Richardson A, Hart MJ, Galvan V (2012). Chronic inhibition of mammalian target of rapamycin by rapamycin modulates cognitive and non-cognitive components of behavior throughout lifespan in mice. Neuroscience.

[56] Tsai PT, Hull C, Chu Y, Greene-Colozzi E, Sadowski AR, Leech JM, Steinberg J, Crawley JN, Regehr WG, Sahin M (2012). Autistic-like behaviour and cerebellar dysfunction in Purkinje cell Tsc1 mutant mice. Nature.

[57] Zhou M, Li W, Huang S, Song J, Kim JY, Tian X, Kang E, Sano Y, Liu C, Balaji J, Wu S, Zhou Y, Zhou Y (2013). mTOR Inhibition ameliorates cognitive and affective deficits caused by Disc1 knockdown in adult-born dentate granule neurons. Neuron.

[58] Johnson SC, Rabinovitch PS, Kaeberlein M (2013). mTOR is a key modulator of ageing and age-related disease. Nature.

[59] Bartels AL, Kortekaas R, Bart J, Willemsen AT, de Klerk OL, de Vries JJ, van Oostrom JC, Leenders KL (2009). Blood-brain barrier P-glycoprotein function decreases in specific brain regions with aging: a possible role in progressive neurodegeneration. Neurobiol Aging.

[60] Chiu C, Miller MC, Monahan R, Osgood DP, Stopa EG, Silverberg GD (2015). P-glycoprotein expression and amyloid accumulation in human aging and Alzheimer's disease: preliminary observations. Neurobiol Aging.

[61] Pekcec A, Schneider EL, Baumgärtner W, Stein VM, Tipold A, Potschka H (2011). Age-dependent decline of blood-brain barrier P-glycoprotein expression in the canine brain. Neurobiol Aging.

[62] Liao CY, Rikke BA, Johnson TE, Diaz V, Nelson JF (2010). Genetic variation in the murine lifespan response to dietary restriction: from life extension to life shortening. Aging Cell.

[63] Liao CY, Rikke BA, Johnson TE, Gelfond JA, Diaz V, Nelson JF (2011). Fat maintenance is a predictor of the murine lifespan response to dietary restriction. Aging Cell.

[64] Zeevi D, Korem T, Zmora N, Israeli D, Rothschild D, Weinberger A, Ben-Yacov O, Lador D, Avnit-Sagi T, Lotan-Pompan M, Suez J, Mahdi JA, Matot E (2015). Personalized Nutrition by Prediction of Glycemic Responses. Cell.

[65] Sohal RS, Ferguson M, Sohal BH, Forster MJ (2009). Life span extension in mice by food restriction depends on an energy imbalance. J Nutr.

[66] Forster MJ, Morris P, Sohal RS (2003). Genotype and age influence the effect of caloric intake on mortality in mice. FASEB J.

[67] Kilkenny C, Browne W, Cuthill IC, Emerson M, Altman DG, National Centre for the Replacement, Refinement and Reduction of Amimals in Research (2011). Animal research: reporting in vivo experiments--the ARRIVE guidelines. J Cereb Blood Flow Metab.

[68] Hong X, To XV, Teh I, Soh JR, Chuang KH (2015). Evaluation of EPI distortion correction methods for quantitative MRI of the brain at high magnetic field. Magn Reson Imaging.

[69] Alsop DC, Detre JA, Golay X, Gunther M, Hendrikse J, Hernandez-Garcia L, Lu H, Macintosh BJ, Parkes LM, Smits M, van Osch MJ, Wang DJ, Wong EC (2014.). Recommended implementation of arterial spin-labeled perfusion MRI for clinical applications: A consensus of the ISMRM perfusion study group and the European consortium for ASL in dementia. Magn Reson Med.

[70] Hartz AM, Bauer B, Soldner EL, Wolf A, Boy S, Backhaus R, Mihaljevic I, Bogdahn U, Klünemann HH, Schuierer G, Schlachetzki F (2012). Amyloid-β contributes to blood-brain barrier leakage in transgenic human amyloid precursor protein mice and in humans with cerebral amyloid angiopathy. Stroke.

[71] Miller DS, Bauer B, Hartz AM (2008). Modulation of P-glycoprotein at the blood-brain barrier: opportunities to improve central nervous system pharmacotherapy. Pharmacol Rev.

